# Barriers and opportunities for female participation in orthopedic surgery: An Indian perspective

**DOI:** 10.6026/973206300211813

**Published:** 2025-07-31

**Authors:** Deepak Kumar, Shah Waliullah, Sanjiv Kumar, Vishal Singh, Sujeet Kumar Chaudhary, Ashish Kumar

**Affiliations:** 1 Department of Orthopaedic Surgery, King George's Medical University, Lucknow, India

**Keywords:** Women in orthopedics, gender bias, career choice, medical education, mentorship, work-life balance

## Abstract

Orthopedic surgery remains one of the least gender-diverse medical specialties in India, with women comprising only about 1% of the workforce. Therefore, it is of interest to explore the barriers that deter female medical students and early-career doctors from pursuing orthopedics, including lack of mentorship, perceived physical demands, gender bias, and concerns over work-life balance. A cross-sectional survey of 250 female trainees across India revealed low interest in orthopedics despite recognition of its financial and career potential. Societal stereotypes and absence of female role models significantly influenced career preferences. Addressing these barriers through structured mentorship, inclusivity in training, and gender-sensitive reforms may improve female representation in orthopedics.

## Background:

Orthopedic surgery has long been viewed as a male-dominated specialty [1]. Historically, the physical demands of the specialty, combined with the perception of gender bias, have deterred female doctors from entering the field [2]. In India, while the number of women in medicine has been steadily increasing, this increase has not been mirrored in surgical specialties, particularly orthopedics [3]. According to the Medical Council of India, only a small percentage of orthopedic surgeons in India are women, which highlights a significant gender gap in this specialty [4]. In India, female orthopedic surgeons constitute approximately 1% of the orthopedic workforce, significantly lower than in other medical specialties [5]. As of December 2022, 369 female orthopedic surgeons were part of the Women's Orthopedic Surgeons of India Collective (WOICE), which aims to support and promote women in this field [6]. In recent years, other male-dominated fields such as engineering, technology, and sports have witnessed increased female participation [7]. Women now top entrance exams at the Indian Institutes of Technology (IITs) and Indian Institutes of Management (IIMs), breaking stereotypes that have long existed in these fields [8].

Just as female athletes like P.V. Sindhu in badminton and Mithali Raj in cricket have broken stereotypes and outshone their male counterparts on the global stage, Indian women in orthopedic surgery are gradually challenging traditional gender norms and proving their capabilities in a historically male-dominated field [9]. These examples show that when systemic barriers are dismantled, women are not only capable but often excel in fields traditionally considered male-dominated [10]. Despite this, orthopedics continues to lag behind [11]. The lack of female mentors in the field, gender bias, and the perception that orthopedic surgery is physically demanding and incompatible with work-life balance are significant factors that deter women from entering the specialty [12]. However, it is crucial to highlight the importance of increasing female participation in orthopedic surgery. Not only will this promote gender equity, but it will also enhance patient care, as studies suggest that diverse teams of surgeons improve clinical outcomes and patient satisfaction [13]. Moreover, female surgeons can bring unique perspectives to patient care, especially for female patients [14]. In areas such as maternal health and osteoporosis management, female orthopedic surgeons can contribute significantly to improving healthcare outcomes [15]. Additionally, female patients may feel more comfortable discussing their health concerns with female doctors, particularly in societies where gender norms impact doctor-patient interactions [16]. Therefore, it is of interest to describe the barriers and opportunities for female participation in orthopedic surgery in India.

## Methodology:

This study was conducted over a two-month period from July to August 2024. A total of 250 participants were included in the study, which comprised final-year MBBS students, interns, and first-year residents. Participants were selected from various medical colleges across India. Data collection was performed using a structured questionnaire prepared in Google Forms, with questions derived from previous studies focusing on factors influencing female doctors' career choices, especially in male-dominated fields such as orthopedics. The questionnaire included both closed and open-ended questions, allowing participants to express multiple perspectives. Key areas covered included demographics, career preferences, perceived barriers to entering orthopedics, and attitudes towards gender roles and work-life balance. The study also focused on the presence or absence of female mentors in the orthopedic departments during medical training and the influence of mentorship on career decisions. The responses were analyzed using descriptive statistics, with a particular focus on identifying trends related to gender-specific barriers and motivators. Results were visualized using pie charts and graphs to highlight key trends.

## Results:

A total of 250 female medical students and early-career doctors participated in the study. The majority (73.8%) were final-year MBBS students, followed by 8.3% interns, with the remaining respondents comprising post-interns and first-year residents. Despite a broad representation of individuals at different stages of medical training, only 9.6% of the participants reported a strong interest in pursuing orthopedic surgery. A significant portion (63.3%) expressed a neutral stance toward the specialty, while the rest were either disinterested or uncertain, suggesting an overall ambivalence or lack of strong inclination among female medical trainees toward orthopedics. When asked about career preferences, 65.5% of respondents indicated that they had never seriously considered orthopedics as a career option. Among the minority who had contemplated it, nearly half (46.7%) reported a primary preference for other specialties such as internal medicine, pediatrics, or obstetrics and gynecology. Qualitative insights revealed a common pattern of early exposure to these other specialties during clinical postings and a perceived lack of exposure or encouragement to pursue orthopedics. Several perceived and experienced barriers were cited as deterrents. Notably, 34.5% of participants pointed to the lack of female mentors in orthopedics as a key reason for their disinterest. This was frequently echoed in the open-ended responses, where participants expressed the difficulty in visualizing themselves in a male-dominated specialty without visible role models. One respondent noted that she had "never seen a female orthopedic surgeon during my rotations," which impacted her belief in the attainability of success in the field. Physical demands emerged as another prominent theme. About 63.3% of respondents considered orthopedics to be "very physically demanding," and an additional 21.4% described it as "extremely demanding." Only 13.1% felt that it was only slightly or not physically taxing. Several respondents conveyed concerns about sustaining long-term physical strain, particularly during pregnancy or while managing family responsibilities in the future. In the qualitative data, three out of five participants described orthopedics as "too physically exhausting," with some even associating it with potential long-term musculoskeletal strain. Gender bias within the specialty was reported by 29.3% of survey respondents, with qualitative responses corroborating this concern. Participants mentioned encountering subtle discouragement or stereotypes implying that orthopedic surgery were "not suitable for women." These attitudes, although often implicit, were powerful enough to influence career choices. Furthermore, 16.6% of respondents indicated that concerns about poor work-life balance deterred them from pursuing orthopedics. This was despite 55% acknowledging that work-life balance was an important factor in selecting any specialty. Interestingly, 51.1% were unsure whether orthopedics could offer a satisfying work-life balance, reflecting a general lack of clarity or guidance on what a career in orthopedics entails in terms of lifestyle. Financial and career-related motivations also played a role in specialty selection. A substantial number of participants (44.5%) rated potential earnings as a "very important" consideration when choosing a career path and 55.9% believed that orthopedic surgery offers high earning potential. Additionally, 47.2% of respondents viewed orthopedics as having favorable long-term career prospects. However, some qualitative respondents expressed uncertainty regarding the initial financial burden associated with setting up a private practice or acquiring surgical skills, especially in resource-limited settings. Societal and cultural factors appeared to exert a significant influence on career aspirations. A large majority (73.8%) acknowledged the presence of societal stereotypes and pressures that tend to discourage women from considering surgical specialties, particularly orthopedics. Despite this, family support was widely reported, with 59.4% of participants stating that their families were "very supportive" of their career ambitions. However, 17.9% admitted that societal expectations played a strong role in shaping their decisions. Participants highlighted the role of gender norms, with some recounting advice from relatives or peers warning them against surgery as a career choice due to the "rough" nature of the work. Qualitative responses further reinforced the survey findings by offering nuanced insights into participants' attitudes. Several respondents emphasized the emotional and psychological toll of navigating a male-dominated field, while others expressed a desire for a more inclusive environment. When asked about potential solutions, participants recommended increasing visibility of successful female orthopedic surgeons, establishing structured mentorship programs, and actively combating gender stereotypes through institutional support and medical education reform.

## Discussion:

This study reveals several key insights into why female medical students and early-career doctors in India are hesitant to pursue orthopedic surgery as a career. The primary deterrents identified in this study align with the findings from previous research, such as the lack of female mentorship and the perceived physical demands of the specialty. Gender bias and discrimination were also significant concerns for many respondents, a finding that mirrors global trends in surgical specialties [17]. However, the study also highlights the potential for increased female participation in orthopedics to bring about positive change. Research suggests that gender diversity in medical teams leads to improved clinical outcomes, and patients often report higher levels of satisfaction when treated by diverse teams. In orthopedic surgery, this could translate to better care for female patients, who may be more comfortable discussing their health concerns with a female surgeon. Moreover, female orthopedic surgeons can bring unique perspectives to areas such as osteoporosis management and rehabilitation, which disproportionately affect women [18]. Increasing the number of female mentors in orthopedics could also have a profound impact. Mentorship plays a crucial role in shaping career choices, and the lack of visible female role models in orthopedics contributes to the perpetuation of gender stereotypes. Establishing mentorship programs and increasing female representation in orthopedic faculties could inspire future generations of female doctors to consider orthopedics as a viable career option [19]. In terms of the benefits for female doctors, orthopedics offers excellent financial rewards and long-term career prospects [20]. The technical complexity and patient outcomes associated with orthopedic surgery can be deeply fulfilling for those who choose to specialize in the field [21]. While physical demands and work-life balance were significant concerns for the participants, the findings of this study suggest that systemic changes-such as creating more flexible work environments and promoting gender equity-could help address these barriers [22].

## Conclusion:

Significant barriers-such as lack of mentorship, gender bias, and physical demands-that deter Indian female doctors from pursuing orthopedic surgery is reported. Addressing these challenges through systemic reforms and inclusive mentorship programs can improve gender diversity in the field. Enhancing female participation in orthopedics will not only advance gender equity but also contribute to better patient care outcomes.
